# Nomogram predicts risk of perineural invasion based on serum biomarkers for pancreatic cancer

**DOI:** 10.1186/s12876-023-02819-y

**Published:** 2023-09-18

**Authors:** Wenbo Zou, Dingguo Wu, Yunyang Wu, Kuiping Zhou, Yuanshu Lian, Gengyun Chang, Yuze Feng, Jifeng Liang, Gao Huang

**Affiliations:** Department of General Surgery, No.924 Hospital of PLA Joint Logistic Support Force, Guilin, 541002 China

**Keywords:** Pancreatic head adenocarcinoma, Surgery, Perineural invasion, Serum indicator, Nomogram

## Abstract

**Background:**

Pancreatic cancer is a fatal tumor, and the status of perineural invasion (PNI) of pancreatic cancer was positively related to poor prognosis including overall survival and recurrence-free survival. This study aims to develop and validate a predictive model based on serum biomarkers to accurately predict the perineural invasion.

**Materials and methods:**

The patients from No.924 Hospital of PLA Joint Logistic Support Force were included. The predictive model was developed in the training cohort using logistic regression analysis, and then tested in the validation cohort. The area under curve (AUC), calibration curves and decision curve analysis were used to validate the predictive accuracy and clinical benefits of nomogram.

**Results:**

A nomogram was developed using preoperative total bilirubin, preoperative blood glucose, preoperative CA19-9. It achieved good AUC values of 0.753 and 0.737 in predicting PNI in training and validation cohorts, respectively. Calibration curves showed nomogram had good uniformity of the practical probability of PNI. Decision curve analyses revealed that the nomogram provided higher diagnostic accuracy and superior net benefit compared to single indicators.

**Conclusion:**

The present study constructed and validate a novel nomogram predicted the PNI of resectable PHAC patients with high stability and accuracy. Besides, it could better screen high-risk probability of PNI in these patients, and optimize treatment decision-making.

## Introduction

Pancreatic cancer are increasingly threatening human life and health and impose a serious disease burden on society worldwide [1]. The inconspicuous early symptoms lead to difficult diagnosis and poor treatment outcome of pancreatic cancer, which increases the mortality rate [2, 3]. To date, radical surgery is the only curable treatment for patients with pancreatic cancer [4], like radical pancreatoduodenectomy (PD) remains the only treatment for pancreatic head adenocarcinoma (PHAC) patients [5]. However, due to the presence of distant metastasis or local invasion, fewer patients are suitable for surgical treatment, and most patients lack surgery opportunity at the time of diagnosis, thus, other treatments like radiotherapy and chemotherapy are the main treatment strategies for pancreatic cancer [6]. Previously, PHAC was reported have a significantly higher incidence compared to the pancreatic body/tail adenocarcinoma [1]. Therefore, improving the prognosis of PHAC had increased our team and numerous researches’ interests.

Perineural invasion (PNI) is considered as a pivotal risk factor of early recurrence and poor prognosis of various malignancies, such as pancreatic [7, 8], biliary tract [9] and colorectal [10, 11] cancer, while the incidence of PNI is very high in PHAC [12]. but mechanism of PNI in tumors is unclear. At present, several studies reveal some potential mechanism mediated the early PNI of pancreatic cancer. Jurcak NR et al. found the axon guidance molecules can promote perineural invasion and metastasis of pancreatic tumors in mice model [13]. Huang et al. also demonstrated the MMP1/PAR1/SP/NK1R paracrine loop contributes to PNI of pancreatic cancer cells [14]. PNI is deemed as a process whereby cancer cells invade the nerves surrounded the tissue, thus causing metastatic spread and pain generation [12], which contributed to the both poor long-term survival and life quality of PHAC patients. Moreover, Fouquet T et al. demonstrated PNI is more accurate than T stage and lymph node status to predict early recurrence after pancreatoduodenectomy for PHAC [15]. Therefore, early identification of PNI is helpful in the management of the patients with PHAC. Up to date, Chari et al. has depicted that 34-40% of patients with pancreatic cancer are associated with diabetes [16], thus, hyperglycemia has certain influence on progression of PHAC. Carbohydrate antigen19-9 (CA19-9) is the most virtual serologic indicator for the diagnosis and predicting prognosis of PHAC [17]. Wang et al. performed a retrospective study to suggest CA19-9 and blood glucose level are novel indicators for neural invasion of PHAC [18]. However, no predictive nomogram was constructed to predict the PNI probability of PHAC patients, and the predictive power of single CA19-9 or blood glucose level reflected the PNI probability was limited, thus, it urgently need to development a novel predictive model to assess the PNI probability accurately.

Therefore, in the present study, we performed the univariate and multivariate logistic regression analyses to screen out the independent risk factors of probability of PNI of patients with PHAC based on a retrospective analysis. Then, a nomogram assessed PNI probability was developed in the training cohort with good predictive accuracy, and verified in the validation cohort. It helps assist clinician to discriminate the patients with high-risk probability of PNI, and further guide the clinical practice.

## Materials and methods

### Patients’ selection

The patients with PHAC who received radical surgery in the Department of General Surgery of the No.924 Hospital of PLA Joint Logistic Support Force between January 2010 and June 2020. The clinical information of these participants was collected and analyzed retrospectively. This study was approved by the Ethics Committee of the No.924 Hospital of PLA Joint Logistic Support Force, and participants’ written informed consent were obtained.

### Inclusion and exclusion criteria

The inclusion criteria: (1) Resectable PHAC; (2) R0 surgery was implemented; (3) PHAC was confirmed by postoperative pathology; (d) American Society of Anesthesiologists (ASA) grade was ≤ III; The exclusion criteria: (1) complications or other causes lead death; (2) incomplete clinical data; (3) accompanied by other malignant tumors; (4) distant metastasis [19].

### Follow-up after resection

After surgery, patients received the following tests every 3–6 months until death or loss to follow-up. Examinations during the follow-up period included CA19-9, chest and abdomen CT, abdomen MRI. The final follow-up date was 30th June 2021.

### Statistical analysis

R software (version 4.0.2) and SPSS software (version 26.0) were used for all statistical analyses and graphics. Main R source packages include “survival”, “rms”, “pROC” [20], et al. Continuous variables were showed as mean (standard deviation) or median (interquartile range), and were tested by Student’s t test or Mann-Whitney U test. Categorical variables were reported as numbers with percentages and tested using Chi-squared test or Fisher’s exact test. The univariate and multivariate logistic regression analyses were used to identify independent risk factors. We used The Youden index and the closest-to-(0, 1) criterion to calculate optimal threshold of nomogram score. The Kaplan-Meier method and the log-rank test was used to depict survival curves. The AUC of the ROC and decision curve analysis (DCA) were employed to estimate the performance of nomogram. All the P value < 0.05 in two-sided was considered statistically significant.

## Results

### Patients’ demographics and clinical characteristics

Of 421 patients underwent radical surgery who were collected for this study, while a total of 389 patients included into this study meet the screen criteria. Based on the 7:3 ratio of distribution, A total of 273 and 116 patients were randomly assigned to the training and validation cohorts. Corresponding detailed demographics and characteristics of PHAC patients in these two cohorts were shown in Table 1, Of note, there were not any differences between the training and validation cohorts (P > 0.05). Comparison of OS and RFS of patients in the two cohorts was no statistical differences (Table 1). Additionally, comparisons of clinicopathological variables among patients with PNI and without PNI are demonstrated in Table 2. Compared with PHAC patients without PNI, these patients with PNI more often had preoperative obstructive jaundice; a higher preoperative hemoglobin, blood glucose, total bilirubin (TBIL), CA19-9, CA125 and fibrinogen (P < 0.05), and the patients with PNI had significantly shorter overall and recurrence free survival than the patients without PNI (P < 0.001).

**Table 1:** Characteristics of patients with pancreatic head adenocarcinoma in the two cohorts (n = 389)

Variables	Training cohort	Validation cohort	*P* value
	(n = 273)	(n = 116)	
Age ≤ 60 years, N (%)	133 (48.7%)	60 (51.7%)	0.587
Male, N (%)	161 (59.0%)	66 (56.9%)	0.704
BMI, mean (SD), kg/m^2^	23.0 (3.0)	23.43 (2.9)	0.153
ASA grade ≤ II, N (%)	233 (85.3%)	107 (92.2%)	0.061
Biliary infection, N (%)	9 (3.3%)	3 (2.6%)	0.960
Obstructive jaundice, N (%)	136 (49.8%)	61 (52.6%)	0.617
Weight-loss ≥ 5 kg, N (%)	34 (12.5%)	9 (7.8%)	0.177
Preoperative Hb, median (IQR), g/L	127.0 (118.0, 137.0)	126.0 (117.0, 138.0)	0.847
Preoperative BG, median (IQR), mmol/L	5.4 (4.8, 6.7)	5.8 (5.0, 7.3)	0.552
Preoperative ALB, median (IQR), g/L	39.1 (36.5, 41.2)	39.0 (35.6, 41.7)	0.864
Preoperative TBIL, median (IQR), µmol/L	28.3 (11.1, 165.5)	29.6 (10.3, 170.4)	0.857
Preoperative CA19-9, median (IQR), U/mL	121.0 (31.4, 372.2)	166.1 (39.3, 401.6)	0.821
Preoperative CEA, median (IQR), ng/mL	3.1 (2.0, 4.5)	2.7 (1.8, 4.8)	0.297
Preoperative CA125, median (IQR), U/mL	12.9 (9.4, 19.8)	13.5 (9.4, 21.3)	0.812
Preoperative FIB, median (IQR), g/L	3.7 (3.1, 4.6)	3.5 (3.0, 4.3)	0.068
Maximum tumor size, median (IQR), cm †	3.0 (2.5, 4.0)	3.0 (2.5, 4.0)	0.629
Median RFS (95% CI), months	14.7 (12.9–16.5)	15.4 (11.3–19.4)	0.607
Median OS (95% CI), months	24.0 (20.7, 29.6)	23.8 (19.3, 28.3)	0.809
Postoperative adjuvant therapy, N (%)	196 (71.8%)	83(71.5%)	0.984

†, preoperative imaging results

Abbreviations: SD, standard deviation; IQR, interquartile range; BMI, Body Mass Index; ASA grade, American Society of Anesthesiologists physical status classification; Hb, hemoglobin; BG, blood glucose; ALB, albumin; TBIL, total bilirubin; CA19-9, carbohydrate antigen19-9; CEA, carcinoembryonic antigen; CA125, carbohydrate antigen125; FIB, fibrinogen; RFS, recurrence-free survival; OS, overall survival; CI, confidence interval

**Table 2:** Clinicopathological characteristics between patients with and without PNI

Variables	PNI-positive group	PNI-negative group	*P* value
	(n = 252)	(n = 137)	
Age ≤ 60 years, N (%)	124 (49.2%)	69 (50.4%)	0.827
Male, N (%)	144 (57.1%)	83 (60.6%)	0.511
BMI, mean (SD), kg/m^2^	23.1 (2.9)	23.1 (2.9)	1.000
ASA grade ≤ II, N (%)	217 (86.1%)	123 (89.8%)	0.297
Biliary infection, N (%)	8 (3.2%)	4 (2.9%)	1.000
Obstructive jaundice, N (%)	149 (59.1%)	48 (35.0%)	**< 0.001**
Weight-loss ≥ 5 kg, N (%)	27 (10.7%)	16 (11.7%)	0.772
Preoperative Hb, median (IQR), g/L	126.0 (117.0, 136.0)	129.0 (119.0, 139.0)	**0.042**
Preoperative BG, median (IQR), mmol/L	5.8 (5.1, 7.3)	5.2 (4.6, 6.1)	**< 0.001**
Preoperative ALB, median (IQR), g/L	38.9 (35.5, 40.8)	39.4 (37.0, 42.1)	0.055
Preoperative TBIL, median (IQR), µmol/L	85.8 (12.6, 210.3)	14.0 (9.3, 67.2)	**< 0.001**
Preoperative CA19-9, median (IQR), U/mL	189.5 (55.8, 569.2)	42.3 (14.1, 206.6)	**< 0.001**
Preoperative CEA, median (IQR), ng/mL	3.2 (2.1, 4.7)	2.6 (1.7, 4.4)	0.484
Preoperative CA125, median (IQR), U/mL	14.0 (9.7, 21.5)	11.7 (8.3, 17.0)	**0.012**
Preoperative FIB, median (IQR), per g/L	3.9 (3.2, 4.7)	3.4 (2.9, 4.3)	**0.001**
Maximum tumor size, median (IQR), cm †	3.0 (2.5, 3.8)	3.0 (2.5, 4.0)	0.275
Median RFS (95% CI), months	12.3 (11.3–13,3)	22.7 (17.6–27.8)	**< 0.001**
Median OS (95% CI), months	19.1 (16.7–21.5)	29.4 (21.9–36.9)	**< 0.001**
Postoperative adjuvant therapy, N (%)	171 (67.9%)	108 (78.8%)	0.356

†, preoperative imaging results

Abbreviations: PNI, perineural invasion; SD, standard deviation; IQR, interquartile range; BMI, Body Mass Index; ASA grade, American Society of Anesthesiologists physical status classification; Hb, hemoglobin; BG, blood glucose; ALB, albumin; TBIL, total bilirubin; CA19-9, carbohydrate antigen19-9; CEA, carcinoembryonic antigen; CA125, carbohydrate antigen125; FIB, fibrinogen; RFS, recurrence-free survival; OS, overall survival; CI, confidence interval

### The prognostic value of PNI for overall survival and recurrence-free survival

**Fig. 1:**
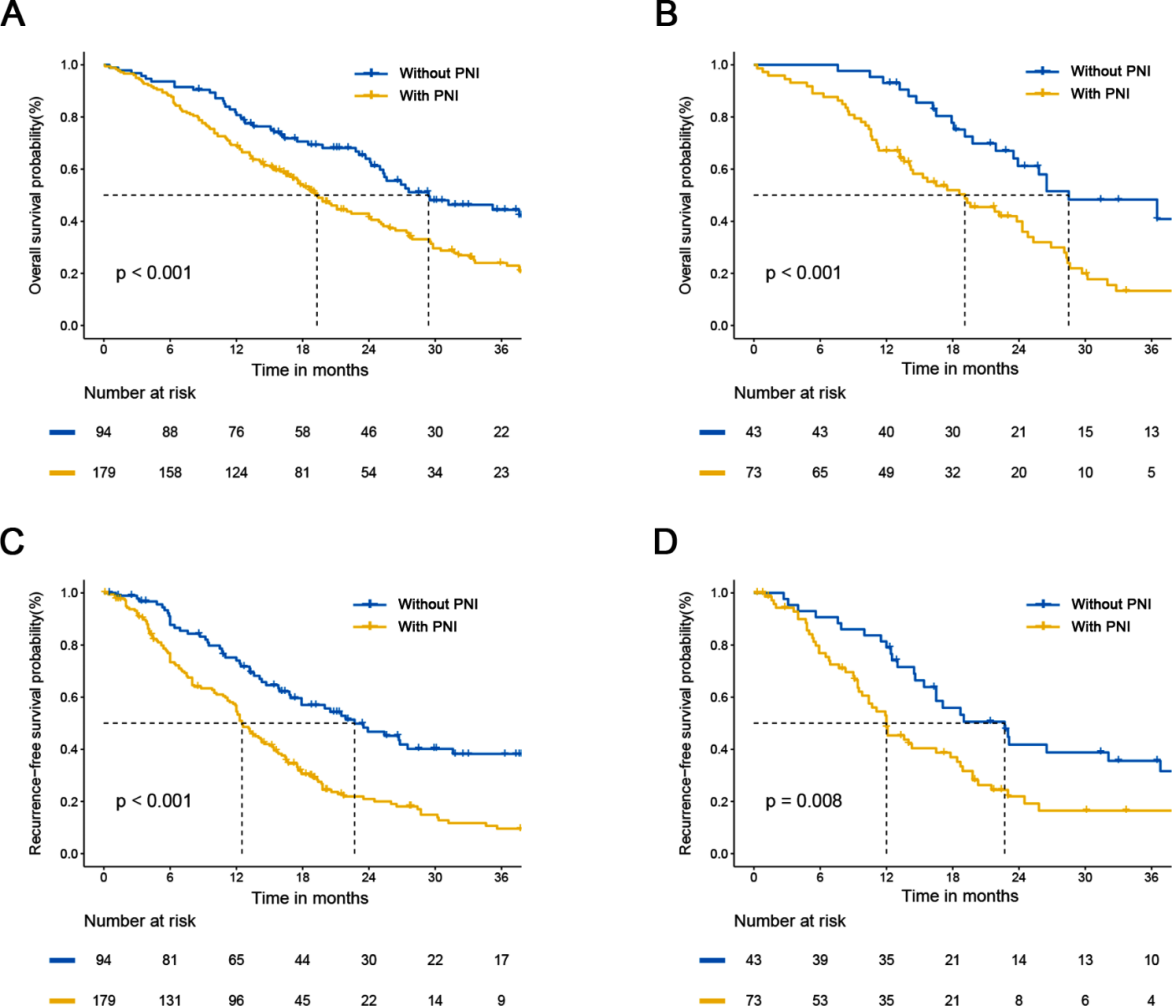
(**A**-**B**) Kaplan-Meier curves of overall survival for patients with and without PNI in the training and validation cohorts. (**C**-**D**) Kaplan-Meier curves of recurrence free survival for patients with and without PNI in the training and validation cohorts

There are significant differences in long-term survival between patients with and without PNI. As shown in Fig. 1A-B, the median OS of patients with PNI was significantly shorter than that of patients without PNI (19.3 vs. 29.4 months and 19.1 vs. 28.5 months) in the training and validation cohorts, respectively (P < 0.001). The median RFS of patients with PNI was also significantly shorter compare to that of patients without PNI (12.5 vs. 22.7 months and 12.0 vs. 22.7 months) in the training and validation cohorts, respectively (Fig. 1C-D, P < 0.001, P = 0.008).

### Identification and selection of independent risk factors for PNI

Univariate logistic regression analysis revealed obstructive jaundice (P < 0.001), preoperative blood glucose (P < 0.001), preoperative albumin (P = 0.034), preoperative total bilirubin (P < 0.001), preoperative CA19-9 (P < 0.001), preoperative CA125 (P = 0.040), preoperative fibrinogen (P < 0.001). Considering the collinearity between the obstructive jaundice and total bilirubin, only the total bilirubin was included in the multivariate analysis (Table 3). Multivariable logistic regression analysis showed preoperative blood glucose (P = 0.001), preoperative total bilirubin (P = 0.029), and preoperative CA19-9 (P = 0.010) were independently risk factors for PNI (Table 3).

**Table 3:** Univariate and multivariate logistic regression analyses for predicting PNI probability of patients with pancreatic head adenocarcinoma in the training cohort

Characteristics	Univariate analysis			Multivariate analysis		
	B	HR (95% CI)	*P* value	B	HR (95% CI)	*P* value
Age, > 60 vs. ≤ 60, years	0.143	1.154 (0.700-1.902)	0.574			
Gender, female vs. male	-0.379	0.685 (0.409–1.147)	0.150			
BMI, per kg/m^2^	0.011	1.011 (0.928-1.100)	0.808			
ASA grade, III vs. ≤ II	0.101	1.107 (0.542–2.261)	0.781			
Biliary infection, yes vs. no	0.051	1.052 (0.257–4.305)	0.944			
Obstructive jaundice, yes vs. no	0.988	2.687 (1.598–4.518)	**< 0.001**			
Weight-loss ≥ 5 kg, yes vs. no	-0.189	0.828 (0.394–1.739)	0.618			
Preoperative Hb, per g/L	-0.015	0.985 (0.968–1.002)	0.084			
Preoperative BG per mmol/L	0.457	1.579 (1.283–1.943)	**< 0.001**	0.336	1.399 (1.144–1.710)	**0.001**
Preoperative ALB, per g/L	-0.073	0.930 (0.869–0.995)	**0.034**	0.010	1.010 (0.932–1.095)	0.808
Preoperative TBIL per µmol/L	0.007	1.007 (1.004–1.010)	**< 0.001**	0.004	1.004 (1.000-1.007)	**0.029**
Preoperative CA19-9, per U/mL	0.002	1.002 (1.001–1.004)	**< 0.001**	0.002	1.002 (1.000-1.003)	**0.010**
Preoperative CEA, per ng/mL	0.017	1.018 (0.962–1.076)	0.541			
Preoperative CA125, per U/mL	0.025	1.025 (1.001–1.050)	**0.040**	0.010	1.010 (0.988–1.031)	0.381
Preoperative FIB, per g/L	0.472	1.603 (1.245–2.064)	**< 0.001**	0.093	1.097 (0.795–1.514)	0.573
Maximum tumor size, per cm †	-0.080	0.923 (0.764–1.115)	0.407			

Bold text hinted that these variables were statistically significant in univariate or multivariate analysis. ¶ Considering the collinearity between the obstructive jaundice and TBIL, only the TBIL was included in the multivariate analysis. †, preoperative imaging results

Abbreviations: HR, hazard Ratio. B, coefficient; CI, confidence interval; BMI, body Mass Index; ASA grade, American Society of Anesthesiologists physical status classification; Hb, hemoglobin; BG, blood glucose; ALB, albumin; TBIL, total bilirubin; CA19-9, carbohydrate antigen19-9; CEA, carcinoembryonic antigen; CA125, carbohydrate antigen125; FIB, fibrinogen

### Development and validation of nomogram predicting probability of PNI

**Fig. 2:**
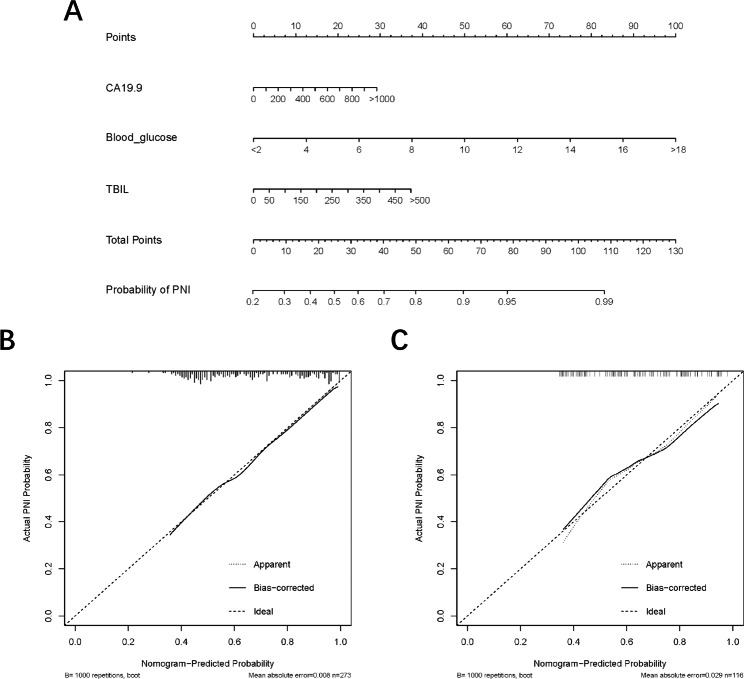
(**A**) The nomogram predicting probability of PNI of patients with PHAC. (**B**-**C**) Calibration curves showing nomogram prediction had the uniformity of the practical probability of PNI in the training and validation cohorts

**Fig. 3:**
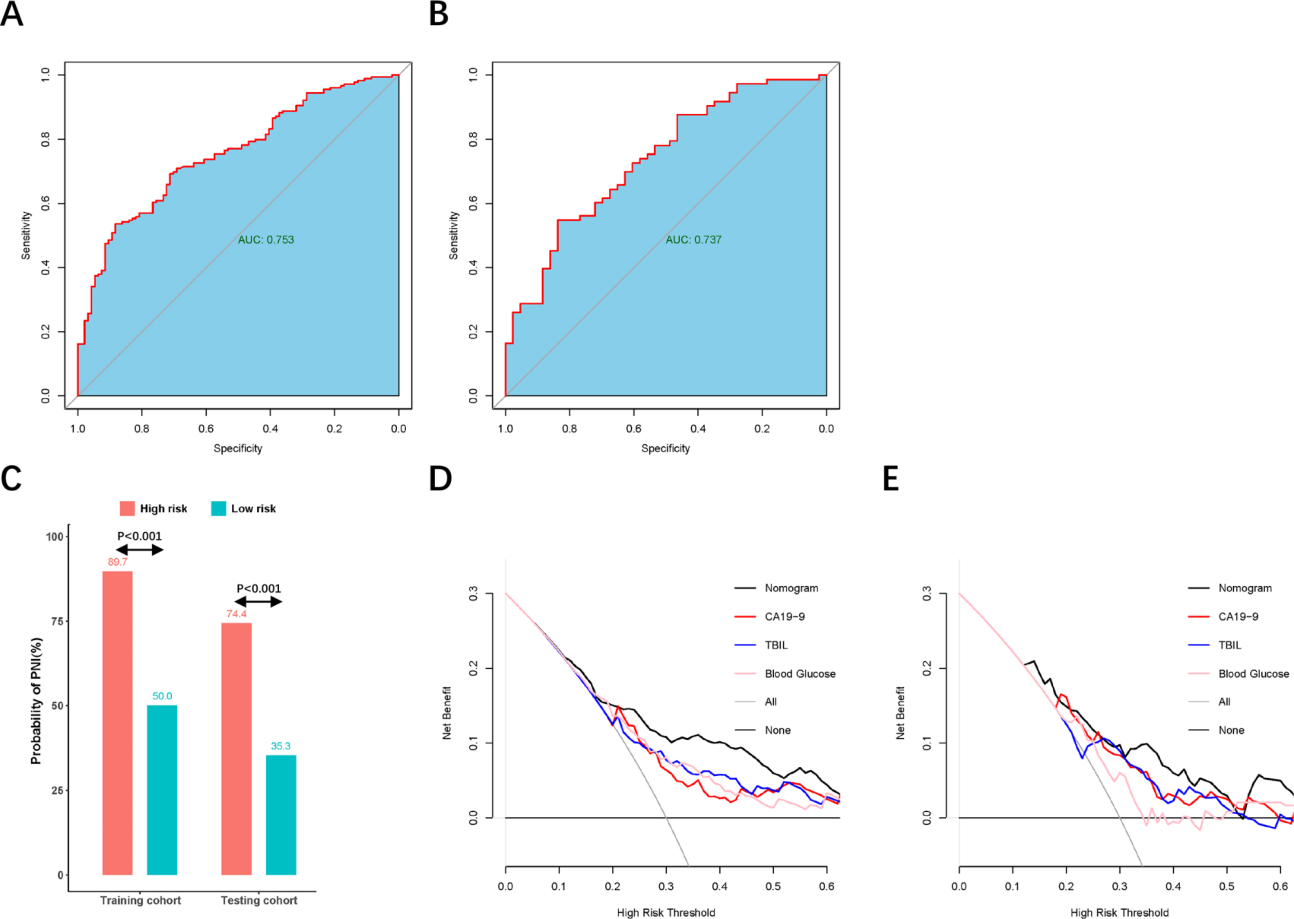
(**A**-**B**) the AUC value of the nomogram prediction in the training and validation cohorts. (**C**) Boxplot of probability of PNI (%) between the high-risk and low-risk in the training and validation cohorts. (**D**-**E**) Decision curve analysis showed clinical benefits of the model predicting PNI compare to the single indicators (CA19-9, total bilirubin, and blood glucose) in the training and validation cohorts

Next, based on all above the independent risk factors, a nomogram was constructed to evaluate PNI probability by summing scores on the point scales for these significant prognostic factors identified in the logistic model (Fig. 2A). The C-statistics of the nomogram was 0.753 and 0.737 in the training and validation cohorts respectively, which showed this nomogram had good predictive capability. The calibration curves for PNI probability showed the nomogram prediction had good uniformity of the practical observation in the training cohort (Fig. 2B), and the validation cohort showed similar results (Fig. 2C). The AUC values for PNI prediction were 0.753 (95% CI, 0.696–0.811), and 0.737 (95% CI,0.645–0.829) in these two cohorts (Fig. 3A-B). The discrimination ability of the nomogram was further evaluated by dividing the predicted probabilities of PNI into two risk groups according to the different nomogram scores (Low-risk group with a nomogram score ≤ 40; and High-risk group with a nomogram score > 40). We found that the high-risk group had a higher probability of PNI than the low-risk group: High risk vs. Low risk, 89.7% vs. 50.0% in training cohort; 74.4% vs. 35.3% in validation cohort (both P < 0.001, Fig. 3C).

### Clinical utility analysis for predictive model

Decision curve analysis (DCA) was used to facilitate comparison between the nomogram models with the other single indicators. The results exhibited more excellent net benefits in predictive model compared with the other single indicators in training and validation cohorts respectively (Fig. 3D-E). It suggested the clinical usefulness of the nomogram was stable and steady in predicting PNI.

## Discussion

The very high mortality and recurrence rate in pancreatic head adenocarcinoma (PHAC) places importance on exploiting the novel predictive model to improve long-term survival of patients with PHAC [1, 4, 21]. At present, several studies showed that perineural invasion (PNI) is considered as the pivotal risk factor for various malignancies including PHAC [10–12]. Thus, it urgently needs a novel nomogram based on some preoperative indicators to predictive PNI probability before surgery. In the present study, we developed a novel nomogram assessed the probability of PNI of patients with PHAC, and validated it in the internal validation cohort. Of 389 patients with PHAC met the include criteria, and plenty of preoperative variables including age, gender, common syndromes, and serum indicators, such as blood glucose, Carbohydrate antigen 125 (CA125), and Carbohydrate antigen19-9 (CA19-9) et al. Based on the univariate and multivariate logistic regression analysis, we finally developed a novel nomogram involved three independent risk factors (preoperative blood glucose, preoperative total bilirubin, and preoperative CA19-9) to predict PNI probability of patients in the training cohort, while these factors were easy to obtain in clinical practice. CA19-9 is the most virtual serologic indicator and predictor for the diagnosis and predicting prognosis of PHAC [17, 22], extremely elevated CA19-9 level often indicate poor prognosis, and it can also play the encouraging role in the progression of PHAC [22]. However, CA19-9 has limited specificity, and thus is not recommended for the early screening of pancreatic cancer. Recently, increasing studies focus on combining multi-serum biomarkers with CA19-9 for pancreatic cancer detection [23]. In addition, the relationship between CA19-9 and PNI was reported by Wang et.al [18], which is similar to our study, the CA19-9 is the independent risk factor of PNI. Like our study, Wang et al. also suggested elevated blood glucose level are positively related with PNI of PHAC [18]. It implies the hyperglycemia take a positive effect on pancreas status and play an accelerated role in malignant progression of PHAC. Therefore, it is essential to surveil the blood glucose level of PHAC patients before surgery timely. Previous study revealed the bilirubin as a risk indicator to evaluate prognosis of PHAC patients [24]. But no study illuminates the correlation of bilirubin with PNI probability. In the present study, preoperative total bilirubin was also considered as an independent risk factor of PNI, and combining it with other two biomarkers can predict the PNI with better predictive accuracy compared to that of single indicator.

The predictive model (nomogram) uses various independent prognostic factors including demographics, biology and genomics to estimate the risk of disease development or potential outcomes [25–28]. It is a valuable tool for healthcare personnel to accurately evaluate the potential risk associated with a specific clinical outcome, allows for informed and confident clinical decision-making [28, 29]. At present, various predictive models were developed and used in personal treatment for cancers including pancreatic cancer. Li et al. constructed a perioperative serum scoring systems predict early recurrence and poor prognosis of pancreatic cancer. The novel serum scoring systems can effectively evaluate the recurrence rate and overall survival rate with good AUC value [30]. Guo et al. developed a nomogram for predicting lymph node positivity in pancreatic head cancer, it has certain ability to predict lymphatic metastasis preoperatively [31]. Besides, some nomogram was developed to predict PNI in diverse tumors [32–34]. Huang et al. constructed a prediction model which integrated the radiomics signature and carcinoembryonic antigen (CEA) level into a prediction model for effective risk assessment of PNI in colorectal cancer [32]. Liu and Huang et al. also developed and validated a nomogram for the preoperative prediction of PNI in patients with gastric and colorectal cancer, respectively [33, 34]. Recently, various clinical-radiomics models were developed to identify the tumor perineural invasion status with good predictive ability. Zhang et al. developed a radiomics nomogram based on multiparametric magnetic resonance imaging for preoperative prediction of perineural invasion status of rectal cancer. They found that the fusion radiomics signature performed better predictive capability to evaluate prognosis [35]. Zhang and Zhan et al. also constructed radiomics model for the preoperative prediction of PNI in patients with prostate cancer and perihilar cholangiocarcinoma, respectively [36, 37]. However, no predictive nomogram was constructed to predict the PNI probability of PHAC patients, and single CA19-9 or blood glucose level reflected the PNI probability was limited, thus, it needs to development a novel predictive model to assess the PNI probability accurately in PHAC patients. In this study, the predictive model we constructed provided a good tool to distinguish patients with high-risk PNI before surgery. Patients with PHAC were preoperatively divided into high-risk group (nomogram score > 40 points) and low-risk group (nomogram score ≤ 40 points). Incidence statistical analyses revealed that patients in the high-risk group had significantly higher incidence than those in the low-risk group. Therefore, the stratification of model could be used for identifying patients who are susceptive to PNI, and further guide the clinician to perform the positive intervention. Next, the results of AUC showed that our model had preferable predictive capability. Decision curve analysis (DCA) was used to facilitate comparison between the nomogram models with the other single indicators [38]. DCA graphically shows the clinical usefulness of the nomogram model for PNI probability on a continuum of potential thresholds for risk of PNI (the x-axis) and the net benefit of using the model to the risk of stratifying patients relative to the assumption that no patient had PNI (the y-axis). In this study, DCA illuminated the model had higher clinical benefits than the single indicators, and the calibration curves also suggested there were good discrimination and calibration capabilities.

Although the present model had a great performance for predicting PNI probability, some limitations also existed. Firstly, the data of all cohorts were collected retrospectively, thus, the study was performed with its inherent defects. Besides, CA19-9 non-secretors were not identified at the baseline and excluded from the study. Secondly, the TNM stage was not obtain before surgery, thus the present nomogram is used for all TNM stage of patients with PHAC. Thirdly, despite the internal validation cohort has been used to increase its reliability, the wide application of this model deserved further confirmation. Therefore, it also needs further large prospective studies to confirm the effectiveness of the present model.

## Conclusion

We developed and validated a novel predictive model assessed probability of PNI of patients with resecable PHAC based on the preoperative serum indicators. Hopefully, this nomogram would help assist clinician to discriminate the PHAC patients with high-risk probability of PNI, and optimize overall treatment decision-making for patients with PHAC.

## Data Availability

The work dataset supports the findings of this study are available on reasonable request from the corresponding author, and the data are not publicly available due to privacy or ethical restrictions.

## References

[1] Mizrahi JD, Surana R, Valle JW, Shroff RT (2020). Pancreatic cancer. Lancet.

[2] McGuigan A, Kelly P, Turkington RC, Jones C, Coleman HG, McCain RS (2018). Pancreatic cancer: a review of clinical diagnosis, epidemiology, treatment and outcomes. World J Gastroenterol.

[3] Zou W, Wang H, Wu D, Wu Y, Zhou K, Lian Y, Chang G, Feng Y, Liang J, Huang G (2023). ncRNA-mediated upregulation of FAM83A is associated with poor prognosis and immune infiltration in pancreatic cancer. Front Endocrinol (Lausanne).

[4] Neoptolemos JP, Kleeff J, Michl P, Costello E, Greenhalf W, Palmer DH (2018). Therapeutic developments in pancreatic cancer: current and future perspectives. Nat Rev Gastroenterol Hepatol.

[5] Delpero JR, Jeune F, Bachellier P, Regenet N, Le Treut YP, Paye F, Carrere N, Sauvanet A, Adham M, Autret A (2017). Prognostic value of resection margin involvement after pancreaticoduodenectomy for Ductal Adenocarcinoma: updates from a french prospective Multicenter Study. Ann Surg.

[6] Stathis A, Moore MJ (2010). Advanced pancreatic carcinoma: current treatment and future challenges. Nat Rev Clin Oncol.

[7] Liu B, Lu KY (2002). Neural invasion in pancreatic carcinoma. Hepatobiliary Pancreat Dis Int.

[8] Ceyhan GO, Demir IE, Altintas B, Rauch U, Thiel G, Müller MW, Giese NA, Friess H, Schäfer KH (2008). Neural invasion in pancreatic cancer: a mutual tropism between neurons and cancer cells. Biochem Biophys Res Commun.

[9] Marchesi F, Piemonti L, Mantovani A, Allavena P (2010). Molecular mechanisms of perineural invasion, a forgotten pathway of dissemination and metastasis. Cytokine Growth Factor Rev.

[10] Horn A, Dahl O, Morild I (1991). Venous and neural invasion as predictors of recurrence in rectal adenocarcinoma. Dis Colon Rectum.

[11] Yang Y, Huang X, Sun J, Gao P, Song Y, Chen X, Zhao J, Wang Z (2015). Prognostic value of perineural invasion in colorectal cancer: a meta-analysis. J Gastrointest Surg.

[12] Bapat AA, Hostetter G, Von Hoff DD, Han H (2011). Perineural invasion and associated pain in pancreatic cancer. Nat Rev Cancer.

[13] Jurcak NR, Rucki AA, Muth S, Thompson E, Sharma R, Ding D, Zhu Q, Eshleman JR, Anders RA, Jaffee EM (2019). Axon Guidance Molecules promote Perineural Invasion and Metastasis of Orthotopic pancreatic tumors in mice. Gastroenterology.

[14] Huang C, Li Y, Guo Y, Zhang Z, Lian G, Chen Y, Li J, Su Y, Li J, Yang K (2018). MMP1/PAR1/SP/NK1R paracrine loop modulates early perineural invasion of pancreatic cancer cells. Theranostics.

[15] Fouquet T, Germain A, Brunaud L, Bresler L, Ayav A (2014). Is perineural invasion more accurate than other factors to predict early recurrence after pancreatoduodenectomy for pancreatic head adenocarcinoma?. World J Surg.

[16] Chari ST, Leibson CL, Rabe KG, Timmons LJ, Ransom J, de Andrade M, Petersen GM (2008). Pancreatic cancer-associated diabetes mellitus: prevalence and temporal association with diagnosis of cancer. Gastroenterology.

[17] Chang JC, Kundranda M. Novel diagnostic and predictive biomarkers in pancreatic adenocarcinoma. Int J Mol Sci 2017, 18(3). 10.3390/ijms18030667 PMC5372679 28335509

[18] Wang PH, Song N, Shi LB, Zhang QH, Chen ZY (2013). The relationship between multiple clinicopathological features and nerve invasion in pancreatic cancer. Hepatobiliary Pancreat Dis Int.

[19] Facciorusso A, Di Maso M, Serviddio G, Larghi A, Costamagna G, Muscatiello N (2017). Echoendoscopic ethanol ablation of tumor combined with celiac plexus neurolysis in patients with pancreatic adenocarcinoma. J Gastroenterol Hepatol.

[20] Robin X, Turck N, Hainard A, Tiberti N, Lisacek F, Sanchez JC, Müller M (2011). pROC: an open-source package for R and S + to analyze and compare ROC curves. BMC Bioinformatics.

[21] Zou W, Wang Z, Wang F, Zhang G, Liu R (2021). A nomogram predicting overall survival in patients with non-metastatic pancreatic head adenocarcinoma after surgery: a population-based study. BMC Cancer.

[22] Luo G, Jin K, Deng S, Cheng H, Fan Z, Gong Y, Qian Y, Huang Q, Ni Q, Liu C (2021). Roles of CA19-9 in pancreatic cancer: Biomarker, predictor and promoter. Biochim Biophys Acta Rev Cancer.

[23] Yang J, Xu R, Wang C, Qiu J, Ren B, You L. Early screening and diagnosis strategies of pancreatic cancer: a comprehensive review. Cancer Commun (Lond) 2021. 10.1002/cac2.12204 PMC8696234 34331845

[24] Imamura T, Okamura Y, Sugiura T, Ito T, Yamamoto Y, Ashida R, Ohgi K, Otsuka S, Uesaka K (2021). Clinical significance of preoperative albumin-bilirubin Grade in Pancreatic Cancer. Ann Surg Oncol.

[25] Zou W, Wang Z, Wang F, Li L, Liu R, Hu M (2021). A metabolism-related 4-lncRNA prognostic signature and corresponding mechanisms in intrahepatic cholangiocarcinoma. BMC Cancer.

[26] Zou W, Zhu C, Wang Z, Tan X, Li C, Zhao Z, Hu M, Liu R (2021). A Novel Nomogram based on log odds of metastatic lymph nodes to predict overall survival in patients with Perihilar Cholangiocarcinoma after surgery. Front Oncol.

[27] Xu S, Zhang XP, Zhao GD, Zou WB, Zhao ZM, Liu Q, Hu MG, Liu R (2023). Derivation and validation of a preoperative prognostic model for resectable pancreatic ductal adenocarcinoma. Hepatobiliary Pancreat Dis Int.

[28] Iasonos A, Schrag D, Raj GV, Panageas KS (2008). How to build and interpret a nomogram for cancer prognosis. J Clin Oncol.

[29] Balachandran VP, Gonen M, Smith JJ, DeMatteo RP (2015). Nomograms in oncology: more than meets the eye. Lancet Oncol.

[30] Li S, Zhang G, Lu Y, Zhao T, Gao C, Liu W, Piao Y, Chen Y, Huang C, Chang A (2022). Perioperative serum Scoring Systems Predict Early recurrence and poor prognosis of Resectable Pancreatic Cancer. Front Oncol.

[31] Guo X, Song X, Long X, Liu Y, Xie Y, Xie C, Ji B (2023). New nomogram for predicting lymph node positivity in pancreatic head cancer. Front Oncol.

[32] Huang Y, He L, Dong D, Yang C, Liang C, Chen X, Ma Z, Huang X, Yao S, Liang C (2018). Individualized prediction of perineural invasion in colorectal cancer: development and validation of a radiomics prediction model. Chin J Cancer Res.

[33] Liu J, Huang X, Chen S, Wu G, Xie W, Franco JPC, Zhang C, Huang L, Tian C, Tang W (2020). Nomogram based on clinical characteristics for preoperative prediction of perineural invasion in gastric cancer. J Int Med Res.

[34] Huang X, Liu J, Wu G, Chen S, Pc FJ, Xie W, Tang W (2019). Development and validation of a Nomogram for Preoperative Prediction of Perineural Invasion in Colorectal Cancer. Med Sci Monit.

[35] Zhang Y, Peng J, Liu J, Ma Y, Shu Z (2022). Preoperative prediction of Perineural Invasion Status of rectal Cancer based on Radiomics Nomogram of Multiparametric magnetic resonance imaging. Front Oncol.

[36] Zhan PC, Lyu PJ, Li Z, Liu X, Wang HX, Liu NN, Zhang Y, Huang W, Chen Y, Gao JB (2022). CT-Based Radiomics Analysis for Noninvasive Prediction of Perineural Invasion of Perihilar Cholangiocarcinoma. Front Oncol.

[37] Zhang W, Zhang W, Li X, Cao X, Yang G, Zhang H. Predicting Tumor Perineural Invasion Status in High-Grade prostate Cancer based on a Clinical-Radiomics Model incorporating T2-Weighted and diffusion-weighted magnetic resonance images. Cancers (Basel) 2022, 15(1). 10.3390/cancers15010086 PMC9817925 36612083

[38] Van Calster B, Wynants L, Verbeek JFM, Verbakel JY, Christodoulou E, Vickers AJ, Roobol MJ, Steyerberg EW (2018). Reporting and interpreting decision curve analysis: a guide for investigators. Eur Urol.

